# The Energy Landscape Perspective: Encoding Structure and Function for Biomolecules

**DOI:** 10.3389/fmolb.2022.820792

**Published:** 2022-01-27

**Authors:** Konstantin Röder, David J. Wales

**Affiliations:** Yusuf Hamied Department of Chemistry, University of Cambridge, Cambridge, United Kingdom

**Keywords:** energy landscapes, multiple functions, biomolecules, folding, disorder, structural heterogeneity

## Abstract

The energy landscape perspective is outlined with particular reference to biomolecules that perform multiple functions. We associate these multifunctional molecules with multifunnel energy landscapes, illustrated by some selected examples, where understanding the organisation of the landscape has provided new insight into function. Conformational selection and induced fit may provide alternative routes to realisation of multifunctionality, exploiting the possibility of environmental control and distinct binding modes.

## 1 Introduction

Since the first work on protein folding over 50 years ago (Levinthal, 1966; Levinthal, 1968; Anfinsen, 1972; Anfinsen, 1973), the advance of new experimental and computational techniques has led to a greatly improved understanding of proteins and nucleic acids. In addition to insights into the structural properties of biomolecules in their functional forms, this work has revealed alternative functional roles. Not only has this research provided a better understanding of the folding process (Karplus, 2011; Wolynes et al., 2012; Wolynes, 2015), but the importance of folding and misfolding in disease has been analysed (Chiti and Dobson, 2017). This improved understanding has enabled the design of new functional biomolecules [for example (Dou et al. 2018), facilitated by the emergence of design principles based on the fundamental principles governing protein folding (Huang et al., 2016; Baker, 2019). While this progress has occurred over a relatively long time scale, the immediate impact was put into focus during the last 2 years, when these techniques were applied to the SARS-CoV2 virus and its molecular constituents to understand structure and function [for example (Bai and Warshel, 2020; Sikora et al., 2021)], to identify potential drugs [for example Shoemark et al. (2021)], and in combination with bioinformatics tools (Waman et al., 2021).

Given the large number of experimental and computational methods commonly used to investigate biomolecules (Röder et al., 2019), it is not surprising that the corresponding results can sometimes seem contradictory and difficult to reconcile. These issues can arise for a variety of reasons. For example, differences in environmental conditions, as well as non-equilibrium effects due to the long timescales associated with biomolecular motions impact both experiment and simulation (Wales and Salamon, 2014). Not only do these effects combine to provide challenges in understanding molecular biological assemblies and their functionality, but they also complicate the design and development of *de novo* biomolecules. Consequently, a viewpoint that allows us to unify and interpret a range of experimental and computational findings is highly desirable.

One key organisational principle emerged from efforts to resolve Levinthal’s paradox, namely the existence of an underlying, funnelled energy landscape (Leopold et al., 1992; Onuchic and Wolynes, 2004; Bryngelson et al., 1995). Starting from the potential energy function, this landscape can be analysed from first principles and contains all the information necessary to describe kinetic, thermodynamic and structural properties of a given molecular system (Wales, 2003). Any method, whether experiment or simulation, samples this energy landscape, but the sampling is often implicit.

The focus of this Perspective involves direct exploration of the potential energy landscape. Here we exploit the natural coarse-grained representation in terms of local minima and the transition states that connect them, which are characterised using geometry optimisation. This framework provides a general description applicable to any molecular system (Wales, 2003). State-of-the-art algorithms allow us to explore such landscapes in the context of a specific set of environmental conditions for a given protein or nucleic acid sequence, and for their complexes. The theoretical insight obtained from simulations can be directly translated into experimental observables; mechanistic explanations for these properties can readily be obtained. This approach elucidates the underlying structural causes for the behaviour of molecular systems, and emergent observable properties, providing both a conceptual and a computational framework (Joseph et al., 2017; Röder et al., 2019). The methodology is briefly summarised in §2, followed by an overview of some recent applications. In particular, we highlight recent analysis of multifunnel biomolecular energy landscapes including intrinsically disordered proteins.

## 2 The Potential Energy Landscape Framework

Our exploration of potential energy landscapes is based on geometry optimisation techniques, and we extract observable thermodynamic and kinetic properties using standard tools of statistical mechanics and unimolecular rate theory (Forst, 1973; Laidler, 1987). There are three distinct but complementary aspects to the calculations. Basin-hopping global optimisation is employed to locate the global minimum and other low-lying structures on the landscape (Li and Scheraga, 1987; Wales and Doye, 1997; Wales and Scheraga, 1999). Here, steps are taken between local minima based on the potential or free energy (Sutherland-Cash et al., 2015) difference and a parameter with dimensions of energy, which determines the probability of accepting an uphill move. Many variants of basin-hopping exist, featuring alternative move sets, accept/reject criteria (Leary and Doye, 1999; Shang and Wales, 2014), and ensembles (Calvo et al., 2016). The common feature that accelerates the exploration is the local minimisation that focuses sampling on a discrete set of structures.

Exploration of the low-lying minima *via* basin-hopping can be coupled with a method such as parallel tempering (Swendsen and Wang, 1986; Geyer, 1991; Hukushima and Nemoto, 1996) that can sample high energy parts of landscape, where the barriers are generally small. This combination provides methods capable of overcoming broken ergodicity problems, such as basin-sampling (Wales, 2013).

### 2.1 Characterising Kinetic Properties Based on Energy Landscape Explorations

Characterising molecular rearrangements and the associated kinetics requires computation of transition states, here defined geometrically as stationary points with a single imaginary normal mode frequency (Murrell and Laidler, 1968). The corresponding algorithms for computing transition states and pathways are relatively mature, and details are available elsewhere (Wales, 2003; Joseph et al., 2017; Wales, 2018; Röder et al., 2019). We refer to pathways defined by local minima and intervening transition states as discrete paths. Having obtained an initial discrete path between target product and reactant states we can expand the resulting database of minima and transition states in various ways to converge the rates of interest (Joseph et al., 2017; Wales, 2018; Röder et al., 2019). The database constitutes a kinetic transition network (Rao and Caflisch, 2004; Noé and Fischer, 2008; Prada-Gracia et al., 2009; Wales, 2010), and we refer to the harvesting of discrete paths as discrete path sampling (DPS) (Wales, 2002; Wales, 2004) by analogy to transition path sampling methods based on explicit dynamics (Bolhuis et al., 2002; Dellago and Bolhuis, 2009). The discrete and dynamical approaches are complementary; the geometry optimisation techniques on which DPS is based are largely agnostic to barrier heights and enable rare events to be analysed on experimental time scales, subject to well-defined approximations.

Post-processing analysis of kinetic transition networks provides rates via mean first passage times, along with a variety of pathway characteristics. The landscapes of interest generally feature highly metastable states corresponding to relatively slow rare events. Extracting observable dynamical properties requires methods based on graph transformation (Trygubenko and Wales, 2006; Wales, 2009; Stevenson and Wales, 2014; MacKay and Robinson, 2018; Swinburne and Wales, 2020), which overcome the numerical problems associated with linear algebra approaches. The first passage time (FPT) distribution can also be analysed, using methods such as kinetic path sampling Athènes and Bulatov, 2014; Athènes et al., 2019), which also exploits graph transformation. This distribution contains far more information than the mean value (Swinburne et al., 2020). For example, we have applied kinetic path sampling to analyse the FPT for tryptophan zipper peptide tryptophan zipper1 (TZ1) (Cochran et al., 2001), revealing a bimodal distribution, which can be directly interpreted in terms of the underlying energy landscape (Sharpe and Wales, 2020). Additional properties such as committor probabilities, reactive visitation probabilities, and productive paths can also be obtained (Sharpe and Wales, 2021a; Sharpe and Wales, 2021b), providing a clear resolution of dynamical bottlenecks.

## 3 The Importance of the Energy Landscape Topography

A key advantage of exploring the energy landscape explicitly is that the organisation is obtained directly. Energy landscapes for proteins and nucleic acids that have evolved to perform a single function are expected to be funnelled (Bryngelson and Wolynes, 1987, Bryngelson and Wolynes, 1989; Leopold et al., 1992; Onuchic et al., 1997; Morcos et al., 2014), allowing fast and reliable folding, and overcoming Levinthal’s paradox (Levinthal (1969)). According to the principle of minimal frustration, the native state is characterised by formation of all native contacts required by the sequence, leading to a single funnel in the potential energy landscape (Bryngelson and Wolynes, 1987; Leopold et al., 1992). However, competing structures might be stabilised in multiple funnels, leading to multifunnel energy landscapes, which are observed for many biomolecules [for example Cragnolini et al., 2017; Röder and Wales, 2018a; Joseph et al., 2019)].

### 3.1 Structural Heterogeneity and Multiple Functions

It is probably not surprising that multifunnelled energy landscapes are commonly observed, since many biomolecules adopt different configurations for alternative functions. Distinct structures require stabilisation to guarantee a sufficient population, since unpopulated structures cannot function. Because such stabilisation needs to be competitive to facilitate control, it leads to separation into distinct ensembles and funnels. Thus, the energy landscape will be multifunnelled. This association between multiple funnels and multiple functions extends the principle of minimal frustration. We expect the energy landscape to support a number of funnels associated with the distinct functions (Röder and Wales, 2018c).

### 3.1.1 Nucleic Acid Landscapes

The energy landscape picture described for proteins is also applicable to nucleic acid folding (Thirumalai and Woodson, 1996; Thirumalai, 1998; Chen and Dill, 2000). However, the structural plasticity is even more pronounced in nucleic acids (Tinoco and Bustamante, 1999; Li et al., 2008; Solomatin et al., 2010), and represents a key challenge in the description of RNA structures (Schroeder, 2018). Nonetheless, the explicit exploration of energy landscapes for nucleic acids is possible, and provides important insight into the structural variation compatible with a given sequence (Cragnolini et al., 2017; Xiao et al., 2019; Röder et al., 2020).

### 3.1.2 Defining Structural Ensembles in Multifunnel Energy Landscapes

While the organisation is revealed by energy landscape explorations, the question remains how to study and analyse the associated, distinct structural ensembles. While energy landscapes are sometimes represented using low-dimensional projections, our approach preserves all degrees of freedom, thus avoiding possible projection errors. The best way to visualise the landscape organisation is using disconnectivity graphs (Becker and Karplus, 1998; Wales et al., 1998), which allow us to identify the funnels on the energy landscape directly. This intrinsic separation of the structures into funnels can also serve as the definition of structural ensembles, where each ensemble is a subgraph of the tree graph representation of the energy landscape. This approach allows us to identify conserved structural motifs within each ensemble, providing a robust definition and analysis of different configurations (Röder et al. 2020).

## 4 Examples

The computational energy landscape framework has been applied for a wide range of biomolecules. This research includes commonly studied systems such as ubiquitin (Röder and Wales, 2018a), key transformations of nucleic acids (Cragnolini et al., 2017; Chakraborty and Wales, 2018; Xiao et al., 2019), smaller disordered peptides (Joseph and Wales, 2018), and biomolecular interactions and binding (Röder et al., 2020). Some of these case studies have been highlighted in previous reviews (Joseph et al., 2017; Röder et al., 2020]. Here, we present two recent examples; the first showing how the methodology can be applied to larger and more complex molecular rearrangements, and the second examining phosphorylation of a protein, and how these chemical modifications modify the landscape.

### 4.1 Influenza a Hemagglutinin Fusion Pathway

Infection by Influenza A is associated with binding of the trimeric hemagglutinin (HA) surface glycoprotein to sialic acid at the termini of glycans in a host cell. First, the HA protein is cleaved into two chains, HA1 and HA2. A key event in the infection pathway is the dissociation of the N-terminal region of HA2 from a helical stem and formation of an extended helix in a “spring-loading” mechanism (Carr and Kim, 1993). The landscape for this process has been characterised to connect pre-fusion (Lin et al., 2012) and post-fusion coiled-coil structures, using a minimal model based on amino acids 33–172 of HA (Burke et al., 2020).

**FIGURE 1:**
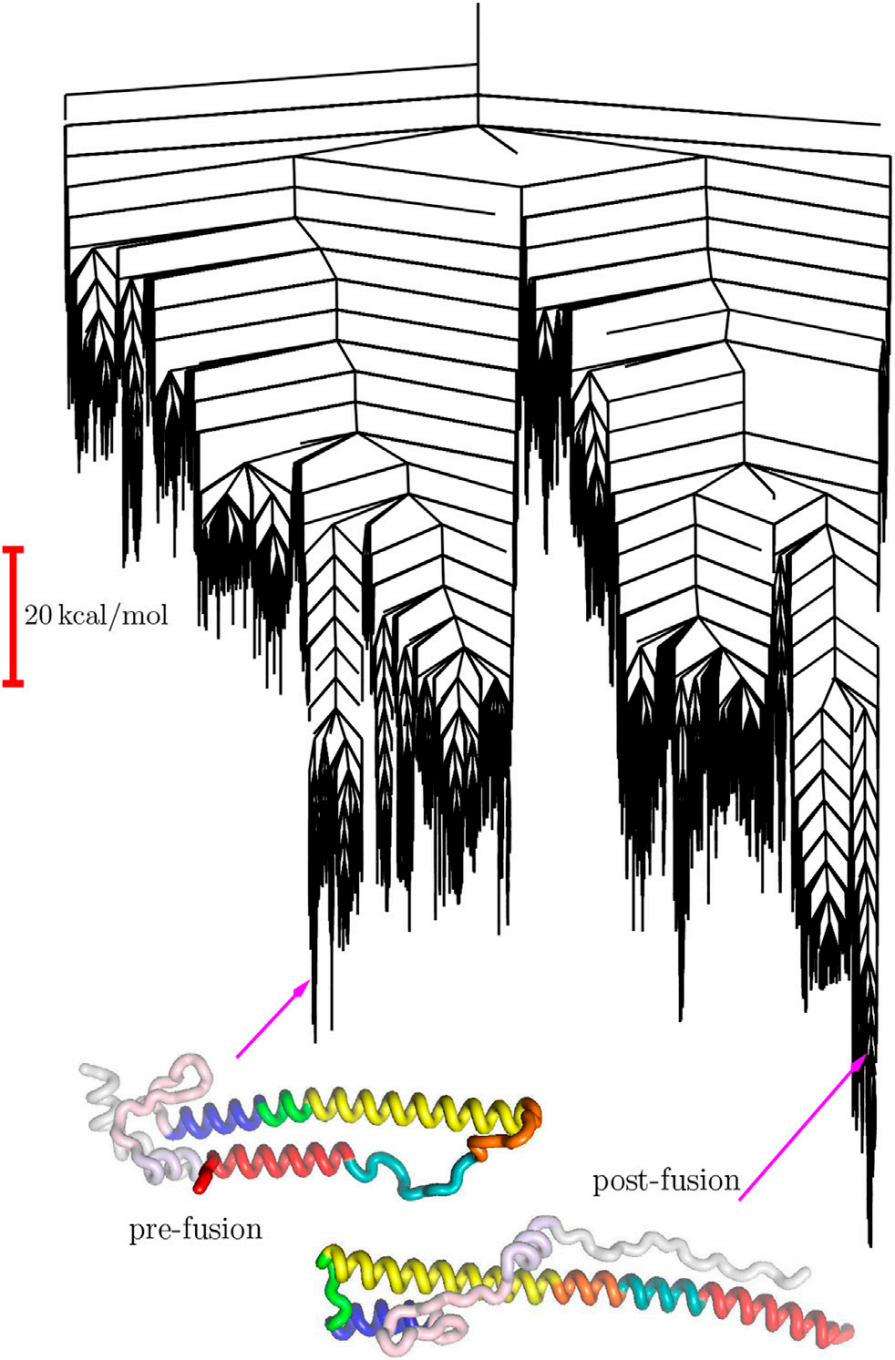
Disconnectivity graph for the HA2 system marking the locations of the pre-fusion and post-fusion minima (Burke et al., 2020).

The predicted pathway involves a two-stage process for conversion of the B-loop into a helical conformation, while the highest barriers are associated with rearrangements that result in reorientation of helical sequences (Burke et al., 2020). The corresponding landscape exhibits a significant range of substructures on various energy and length scales. At the most coarse-grained level there are two principal funnels, associated with the pre- and post-fusion structures, separated by a high barrier (Figure 1). This overall structure is consistent with a switch, with an essentially irreversible transformation on the relevant experimental time scale.

### 4.2 A Phosphorylated Protein

Phosphorylation can induce significant changes in the relative stabilities of alternative protein conformations, and hence change the organisation of the energy landscape. Such changes have been seen for a variety of intrinsically disordered proteins, suggesting that reorganisation may be associated with specific stabilisation of marginally metastable states and the associated funnels in the energy landscape. We have investigated one particular example, namely eukaryotic translation initiation factor 4E (eIF4E) binding protein 2 (4E-BP2), a 120-residue system that exhibits local secondary structure (Fletcher et al., 1998). In the unphosphorylated state, 4E-BP2 competes with eukaryotic translation initiation factor 4G (eIF4G) for binding to eIF4E, and the resulting complex exhibits *α*-helical structure (Fukuyo et al., 2011). Phosphorylation can occur at up to five sites, and stabilises a four-stranded *β*-sheet structure, with low affinity for eIF4E (Bah et al., 2015). The balance of stability is shifted to the *β*-sheet upon phosphorylation at two sites, but in this state the protein retains some affinity for eIF4E. This balance is also strongly affected by mutations.

To understand these effects we explored the energy landscapes of the doubly phosphorylated wild type (pWT) protein and two mutants, p(D33K) and p(Y54A/L59A) (Kang et al., 2020). All the potential and free energy landscapes exhibit multifunnel organisation, with four states competing with the folded conformation in pWT. Differences in relative stability can be interpreted in terms of missing hydrogen-bonds, and the p(D33K) landscape exhibits the most frustration, with four states of similar stability separated by high barriers. Some of the states with minority equilibrium populations in pWT include *α* helical structure in the binding motif, which would explain the residual affinity for eIF4E, and could account for NOE signals that do not arise from the dominant folded conformations. The minority states also feature stabilisation from hydrogen-bonds to the two phosphate groups. Hence phosphorylation causes reorganisation of the multifunnel landscape, which appears to be associated with control of binding affinity functionality. This changes is reminiscent of the reorganisation caused by mutations (Röder and Wales, 2018c), and can also be observed in nucleic acids (Sharpe et al., 2020); Röder et al., 2021).

## 5 Intrinsically Disordered Systems

While globular proteins associated with a single function are expected to exhibit a single funnel landscape, a multifunnel organisation seems necessary to support additional structural ensembles, and hence funnels, required to fulfil distinct functions. In contrast to the energy landscapes characterised for structural glass-formers (de Souza and Wales, 2008; Niblett et al., 2016; de Souza and Wales, 2016), we expect to see a relatively small number of well-defined funnels in such cases. The question then arises: where on this continuous spectrum of energy landscape topographies do we find intrinsically disordered proteins? Descriptions in terms of glassy landscapes may arise from the observation of structural heterogeneity and kinetic trapping in experiments. Due to those features, the experimental and computational study of such systems is more complicated (Baker and Best, 2014; Bhattacharya and Lin, 2019); Strodel, 2021). It is noteworthy that the same observations may be made about non-coding, but functional, RNAs, which also exhibit structural heterogeneity and kinetic trapping. However, these system are rarely described as disordered.

### 5.1 Distinct Energy Landscape Topographies Exist for IDPs

Further questions arise when we look closer at energy landscapes for intrinsically disordered systems. A NMR study of the Nuclear Coactivator Binding Domain, an intrinsically disordered system, revealed the existence of two distinct states, resembling fold-switch proteins (Kjaergaard et al., 2013). In contrast, the free energy landscape of the pKID region of the cAMP response element-binding (CREB) protein exhibits a shallow funnel, allowing for structural heterogeneity, while upon binding the funnel becomes steeper, leading to well defined structure (Chong and Ham, 2019). Systems described as intrinsically disordered proteins and peptides therefore fall into at least two categories: namely molecules that exhibit a number of competing states, where no state is thermodynamically preferred, and those exhibiting a shallow energy landscape, with no or little topographic bias.

**FIGURE 2:**
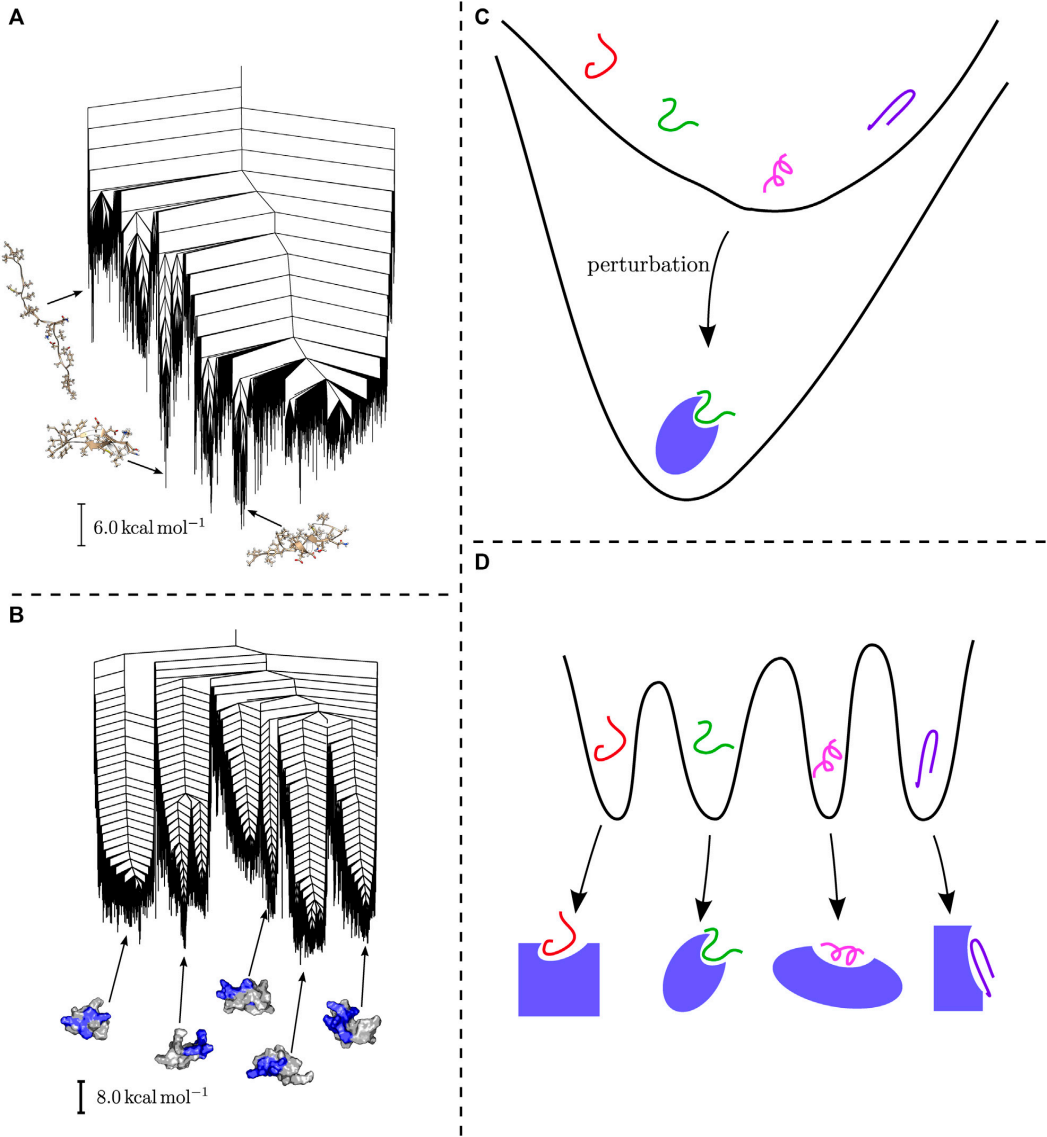
**(A)** The disconnectivity graph for the energy landscape of an amyloid-*β* monomer adapted from (Röder and Wales, 2018b) exhibits a shallow landscape with small subfunnels, while **(B)** the landscape for the H4 histone tail, adapted from (Röder, 2021), supports multiple funnels with distinct structural ensembles. **(C)** Schematic representation of how binding may affect shallow energy landscapes. **(D)** Conformational selection based on a multifunnel energy landscape allows binding to alternative partners for a single peptide or protein.

This observation has a number of important consequences. Firstly, in both cases the energy landscape might be altered through external influences, promoting a subset of dominant structures. Secondly, the existence of multifunnel energy landscapes, which are associated with controlled biological function, might hint at an evolutionary development towards specific sequences. Which type of energy landscape is exhibited is dependent on the sequence and the resulting interactions, and can be discussed in these terms (Uversky, 2019). Importantly, these structural preferences lead to differences in binding behaviour, as observed in experiment (Arai et al., 2015). The two binding mechanisms described in this context are induced fit and conformational selection. It is straightforward to correlate these mechanisms with the organisation of the energy landscape. Induced fit relies on flexibility and adaptation of the structure to external drivers, i.e. the structure of the binding partner, and is likely associated with the shallow energy landscapes. The induced fit then restores the topographical bias through the alteration of the folding funnel (Arai et al., 2015; Chong and Ham, 2019). In contrast, multifunnel energy landscapes seem likely to be associated with conformational selection, with specific interactions stabilising the alternative structural ensembles. Schematic representations of these two ideas are shown in Figure 2.

This picture is supported by results from the explicit exploration of energy landscapes, which yield important insight into the corresponding organisation and structural ensembles (Chebaro et al., 2015); Joseph and Wales, 2018). For example, the amyloid-*β* monomer exhibits a shallow folding funnel, with some minor substructure, and significant structural heterogeneity (Röder and Wales, 2018b). In contrast, the energy landscape for the H4 histone tail exhibits multiple funnels, with essentially identical energies, but widely varying associated structural ensembles (Röder, 2021). Disconnectivity graphs for both examples are shown in Figure  2.

Aggregates of misfolded proteins are likely to be associated with high energy barriers and a more frustrated energy landscape (Strodel, 2021; Röder and Wales, 2018b). This observation is perhaps not surprising, given the large number of possible interactions for even two or three peptides with structural heterogeneity. However, this class of energy landscape should be distinguished from the structure expected for IDPs, which arise from different considerations. This distinction is important considering the association of multiple funnels and multiple functions. Amyloid aggregation is a process that probably has not been challenged by evolution, as it only occurs late in life.

## 6 Machine Learning and Energy Landscape Exploration

Here we briefly consider the future potential of machine learning methods for exploration of energy landscapes and hence contributing to this perspective. Structure prediction based on knowledge-based approaches can now locate low energy conformations efficiently given appropriate data (Tunyasuvunakool et al., 2021); some protein and peptide targets, especially disordered systems, are not yet accessible (Strodel, 2021). Predicting a single structure is not sufficient for a survey of the landscape, as many biomolecules exhibit multiple structural ensembles, which define multiple functionality. However, identifying the structures that underpin multifunnel landscapes may be a realistic target for data driven methods, and would provide input for pathway analysis employing geometry optimisation techniques, as described above. Machine learning also has the potential to accelerate some of this geometry optimisation directly (Garrido Torres et al., 2019); Yang et al., 2021). The prediction of nucleic acid structures represents a further ongoing challenge.

Important advances have also been made in the application of ML-based methods throughout the field of protein simulations (Noé et al. (2020)). Examples include efficient and more accurate potentials [for example Smith et al. (2017)], design of coarse-grained potentials (Wang et al. (2019)), and work on general frameworks to represent atoms and molecules based on the underlying physics (Schütt et al., 2018; Musil et al., 2021). In addition, subsequent analysis steps, for example the calculation of kinetic properties, can also benefit from ML-based frameworks (Mardt et al., 2018; Olsson and Noé, 2019). Such advances should provide more accurate physically motivated models. The bottleneck for most current simulations, especially those that aim to explore large parts of the energy landscape, is the high computational cost. Faster and more accurate methods will find widespread applications for enhanced sampling (Ribeiro et al., 2018; Noé et al., 2019) and treatment of high-dimensional systems (Olsson and Noé, 2019), with new physical insight beyond structure prediction.

## Data Availability

The original contributions presented in the study are included in the article/Supplementary Material, further inquiries can be directed to the corresponding author.
